# Fitness-Dependent Effect of Acute Aerobic Exercise on Executive Function

**DOI:** 10.3389/fphys.2019.00902

**Published:** 2019-07-10

**Authors:** Lin Li, Shu Zhang, Jie Cui, Li-Zhen Chen, Xiaoyan Wang, Mingxia Fan, Gao-Xia Wei

**Affiliations:** ^1^Key Laboratory of Adolescent Health Assessment and Exercise Intervention of Ministry of Education, East China Normal University, Shanghai, China; ^2^School of Physical Education and Health Care, East China Normal University, Shanghai, China; ^3^Key Laboratory of Behavioral Science, Institute of Psychology, Chinese Academy of Sciences, Beijing, China; ^4^Department of Psychology, University of Chinese Academy of Sciences, Beijing, China; ^5^School of Teacher Education and Psychology, Sichuan Normal University, Chengdu, China; ^6^Shanghai Key Laboratory of Magnetic Resonance, East China Normal University, Shanghai, China

**Keywords:** fitness, acute exercise, neural correlates, functional magnetic resonance imaging, cognitive function

## Abstract

Cognitive gains are reported to be induced by acute aerobic exercise, but the role of fitness in the effect of acute aerobic exercise on executive function remains unknown. Therefore, we aimed to examine the effect of fitness on acute exercise-induced changes in executive function from neural mechanism approach. Twenty-four female college students were assigned to high-fitness or low-fitness groups based on their cardiovascular fitness level, and then underwent functional magnetic resonance imaging while performing N-back tasks before and after 30 min of acute exercise. The behavioral results revealed significant interaction effects of group by time in the 0-back and 1-back tasks, but not in the 2-back task. The accuracy was significantly higher in the high-fitness group than in the low-fitness group before exercise in the 1-back and 2-back tasks. At the neural level, significant interaction effects of group by time were observed in all tasks. The 0-back and 1-back tasks activated the right cerebellum while the 2-back task activated subcortical regions. Our findings suggest that fitness moderates the effect of aerobic exercise on cognitive function, and provide the first neural evidence to support the influence of fitness on exercise-induced cognitive performance.

## Introduction

A growing body of literature has documented that a single session of aerobic exercise could improve cognitive performance (Hillman et al., 2008), which could not only be observed in children and adolescents (Hillman et al., 2009; Hogan et al., 2015), but also in older adults (Chang et al., 2015). Further studies have demonstrated that the cognitive benefits gained from acute exercise included extensive information processing categories such as attention, memory, and executive function (Li et al., 2014; Etnier et al., 2016; Hung et al., 2016; Metcalfe et al., 2016). Colcombe and Kramer (2003) reviewed fitness effects among older adults in a meta-analysis and reported that aerobic exercise had robust but selective benefits on cognition, with the largest fitness-induced benefits occurring for executive-control processes. Executive function is a set of cognitive processes including working memory, inhibition, and shifting, which play an essential role in controlling and regulating advanced cognitive process toward targeted behavior (Funahashi, 2001). Hence, it is of great significance to investigate acute exercise-induced executive function for its dominance in human cognitive function with regard to the mental health and social development of individuals (Diamond, 2013).

Cardiovascular fitness refers to the efficiency with which the heart pumps blood and oxygen to the body. It is a profile of the physical ability to supply oxygen-rich blood to the working muscle tissues and the ability of the muscles to use oxygen to produce energy for movement, which is an important determinant of global health throughout the lifespan. Poor cardiovascular fitness is associated with higher illness and health problems from many causes including cardiovascular disease and cancer (Carnethon et al., 2005). Notably, some studies observed differences in executive function between high-fit individuals and low-fit individuals following acute aerobic exercises (Buck et al., 2008; Hogan et al., 2013; Chang et al., 2015; Chu et al., 2017). Prospective studies with large samples also provided substantial evidences demonstrating the association between cardiovascular fitness and improved cognition across ages. Three hundred and forty-nine older adults who were assigned into three different fitness level groups based on their peak maximal oxygen consumption (VO_2_) for 6 years and observed significant group differences in cognitive ability including executive function, verbal memory, and verbal fluency. The high fitness group performed better than the medium and low fitness groups in all cognitive tests. Poorer baseline cardiorespiratory fitness was related to poorer performance on all cognitive tasks even 6 years later (Barnes et al., 2003).

Recently, studies have examined whether fitness moderates the association between acute exercise and executive function. Chang et al. (2014) emphasized the role of cardiovascular fitness in the effect of aerobic exercise on cognitive performance, and detected a curvilinear relationship between fitness and task performance. Researchers have suggested that the association between exercise and executive control is affected by individual differences in fitness level (Colcombe and Kramer, 2003; Kramer and Colcombe, 2018). However, a recent meta-analysis examined the moderating effect of aerobic fitness on the exercise-induced benefits on executive function, and failed to detect that the significant effect of moderate aerobic exercise on time-dependent measures of executive control differs when categorizing participants as low-fit, average-fit, and high-fit (Ludyga et al., 2016). Therefore, the role of fitness in moderation of the association between acute exercise and executive function is largely unknown. In view of the findings regarding aerobic fitness and executive function are generally mixed in behavioral level, it is plausible to clarify such relationship if different pattern of neural activity was detected between high-fit individuals and low-fit individuals when performing the same task of executive function. Therefore, in this study, we employed functional magnetic resonance imaging (fMRI) to examine the effect of fitness on acute exercise-induced changes in executive function. We hypothesized that significant differences will be observed in the executive function and brain activation patterns between high-fitness and low-fitness individuals after acute aerobic exercise.

## Materials and Methods

### Participants

Fifty right-handed healthy female college students were recruited from East China Normal University by flyers and websites. The inclusion criteria were as follows: (1) those with normal or corrected-to-normal vision without color blindness; (2) those who were neither menstruating nor pregnant; and (3) those who were free of internal metal objects or implants. All participants were screened using the Health Screening Questionnaire (HSQ) and Physical Activity Readiness Questionnaire (PAR-Q) to confirm that they could safely participate in the cardiovascular fitness test. Those with a history of physical and brain injury seizures, systematic neurological disease, drug or alcohol abuse, and any other illnesses were excluded from this study. In addition, the participants were also asked not to consume alcoholic or caffeinated beverages and to have a sufficient sleep within 24 h before formal treatment. The participants provided written informed consent and received compensation of 100 RMB after the experiment. The study protocol was approved by the Institutional Review Board of East China Normal University and met the standards of the Declaration of Helsinki.

Eligible participants who met the inclusion criteria were then assigned to either the high-fitness group (*n* = 12) or the low-fitness group (*n* = 12) based on their maximum oxygen consumption (VO_2max_) as determined by cardiovascular fitness assessment. The participants were categorized based on the 50% VO_2max_ cut-off value.

### Experimental Procedure

**FIGURE 1:**
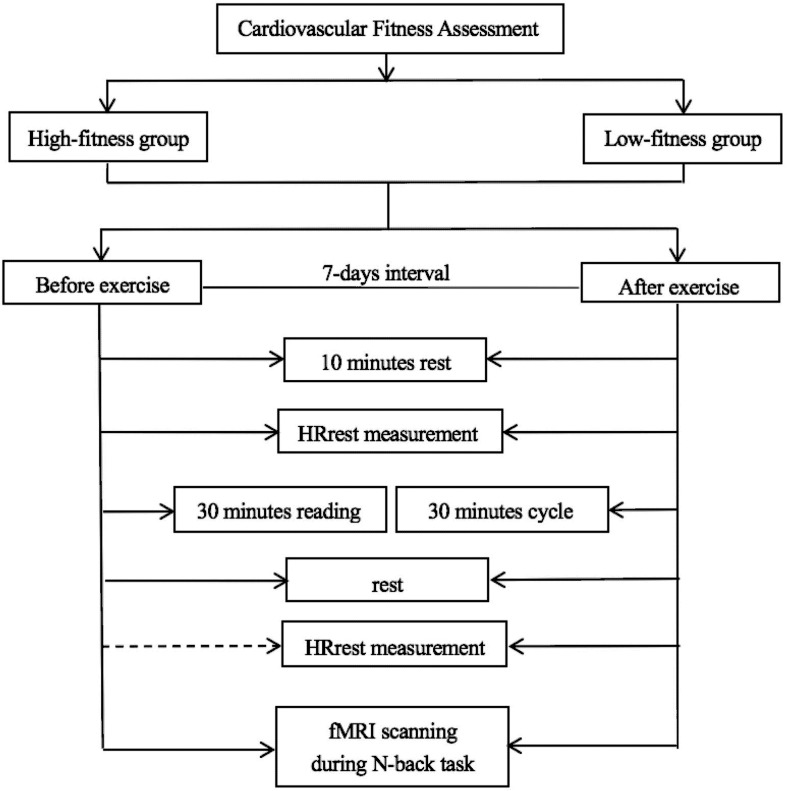
Experimental procedure involving group assignment and exercise intervention.

The procedure is depicted in Figure 1. All participants were requested to visit the laboratory three times. During their first visit, the participants first completed demographic forms and questionnaires, and then completed the assessment of cardiovascular fitness.

The next two visits involved the interventions, which were separated by a 7 day interval. The two interventions occurred at the same time of day for both visits. To minimize the practice effect, a counterbalanced order design was applied. The participants underwent a physical education course for 30 min on 1 day and exercise intervention on the other day.

### Cardiovascular Fitness Assessment

Each participant completed an indirect maximal oxygen uptake (VO_2max_) test on a cycle ergometer in order to measure the individual’s cardiovascular fitness based on the YMCA protocol (Golding, 2000). Before the fitness test, the participant was asked to rest for 10 min, during which their age, gender, weight, height, and resting heart rate (HR) were recorded. The participant then underwent one session of ergometer cycling. The intensity of exercise was set at 50 rpm for 6–9 min. After the fitness assessment, the participants were assigned to either the high-fitness group (24–19 ml/kg/min) or the low-fitness group (17–21 ml/kg/min) based on the median VO_2max_ after excluding the outliers.

### Cognitive Task

**FIGURE 2:**
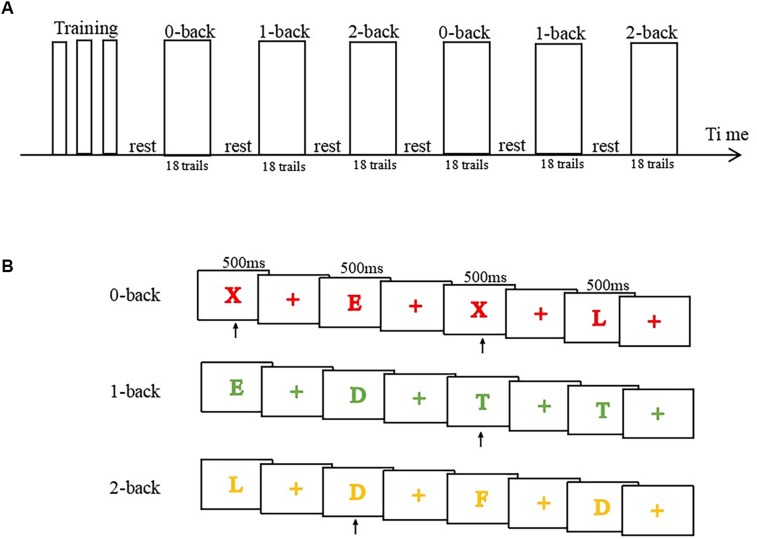
Illustration of the experimental procedure of n-back tasks during fMRI. **(A)** Task procedure and time arrangement using a block design. **(B)** Illustration of four trials of the 0-back, 1-back, and 2-back tasks.

The N-back task was presented using E-prime 2.0 developed by Psychology Software Tools, Inc. software in order to evaluate executive function. The participants were instructed to determine whether the present stimulus was consistent with the previous one presented N (representing cognitive load and is typically equal to 1, 2, or 3) steps before the target in a sequence of letter stimuli. The task consisted of six blocks (18 trials per block) of alternating 0, 1-, and 2-back conditions. Each block involved only one condition. In each trial, the stimulus (letter) was randomly presented at the center of the screen for 500 ms, which was preceded by a fixation cross (1,500 ms). The participant was requested to respond within 2000 ms according to the instruction of cognitive load. During the 0-back condition, the participants were asked to indicate if the currently presented letter was the same or different from the target letter. During the 1-back condition, the participants were requested to indicate if the currently presented letter was the same or different from the letter immediately preceding it. During the 2-back condition, the participants were requested to indicate if the currently presented letter was the same as or different from the letter presented two steps earlier. During all conditions, the participants were instructed to respond as quickly and accurately as possible by pressing the button with the thumb of the right hand. Before the formal experiment, the participants were asked to practice the trials in all conditions until they were familiar with the task performance (Figure 2).

Visual stimuli were displayed using MRI compatible NNL goggles (NordicNeuroLab, Bergen, Norway). Behavioral responses including reaction time and accuracy were collected using MRI-compatible NNL response grips (Nordic NeuroLab, Bergen, Norway), which were connected and held in the right hand.

### Functional Magnetic Resonance Imaging

Participants underwent fMRI twice, which was conducted in two rounds. Before each fMRI scan, the participants were requested to rest for 20 min in a quiet room in order to maintain their HR at <85 bpm. Brain imaging was performed on a 3T Trio system (Siemens, Magnetom, Germany) with a 32-channel head matrix coil. High resolution anatomical images were acquired using a magnetization prepared rapid gradient echo (3D MPRAGE) with repetition time (TR) = 2530 ms, echo time (TE) = 2.34 ms, inversion time (TI) = 1100 ms, flip angle (FA) = 7°, thickness = 1 mm, field of view (FOV) = 256 mm × 256 mm, and voxel size = 1.0 mm × 1.0 mm × 1.0 mm. An echo planar imaging (EPI) sequence was used to obtain functional images, with TR = 2000 ms, TE = 30 ms, FA = 90°, slice thickness = 3.75 mm, matrix = 64 × 64, voxel size = 1.0 mm × 1.0 mm × 1.75 mm, and FOV = 192 mm × 192 mm with 32 axial slices.

### Exercise Intervention

Regarding the exercise intervention, the participants began with a 5 min warm-up exercise on a cycle ergometer after 10 min rest in a quiet room when they arrived. During the warm-up period, they were required to maintain their HR at approximately 120 bpm. Then participants then underwent 20 min of aerobic exercise and maintained their HR within the target range. The individual target HR was calculated by the Karvonen formula: Target HR = ([HR_max_ - HR_rest_] × %intensity) + HR_rest_. In addition, the maximum HR could be estimated using the traditional formula: 220 minus the age. Further, the intensity of the aerobic exercise in this study was set at 60–69%, which was regarded as moderate intensity. The exercise protocol also included a 5 min cool-down period; therefore, the exercise intervention was performed for 30 min in total. Brain imaging was then performed when the participants conducted N-back task within 20 min after the cessation of exercise. HR was also recorded for each participant after exercise. A similar experimental procedure was used for the physical education course, including fMRI and HR recording, but did not involve exercise intervention.

### Statistical Processing and Analysis

Functional magnetic resonance imaging data were analyzed using SPM12^1^. The first 12 EPI images of each time series were discarded to allow for T1 stabilization. All images of head movement were less than 3 mm and 3°. For each participant, echo planar images were slice-time corrected and realigned to the first image, followed by normalization to the standard Montreal Neurological Institute EPI template with voxel resample = 3.0 mm × 3.0 mm × 3.0 mm and spatial smoothing using an 8 mm^3^ full width at half maximum Gaussian kernel. Contrast images were generated for each participant for the contrasts of interest (0-back > rest, 1-back > rest, 2-back > rest, respectively, in two treatments), representing the pair-wise comparison of parameter estimates for the conditions, and separate single-group *t*-tests were then used to identify within-group contrasts of interest. Furthermore, a two-sample *t*-test was performed to examine the differences between two groups with an uncorrected cluster significance threshold of *p* = 0.001 and a cluster size of >10 voxels.

Demographic and behavioral data were analyzed using SPSS 20.0. An independent *t*-test was used to analyze the differences between the high-fitness and low-fitness groups. A repeated measures ANOVA in a general linear model was adopted to examine within-group differences. *Post hoc* analysis was also used to investigate the differences between N-back conditions.

## Results

### Participant Characteristics

No significant differences were found in age, education, height, weight, and body mass index between the high-fitness group and the low-fitness group (Table 1). Additionally, the resting HR did not differ between the high-fitness group and the low-fitness group. However, there was a significant difference in the VO_2max_ between the two groups (*t* = 10.79; *p* < 0.001). This indicated that high-fitness group had higher cardiovascular fitness than that of the low-fitness group, which confirmed the appropriate grouping of the participants in this study based on the fitness level.

**TABLE 1:** Descriptive data of high-fitness group and low-fitness group (mean ± SD).

	**High-fitness**	**Low-fitness**	***p***
*N*	12	12	-
Age (years)	25.50 ± 0.67	25.75 ± 0.62	0.36
Height (cm)	164.75 ± 5.93	161.08 ± 4.88	0.11
Weight (kg)	55.67 ± 5.12	52.00 ± 4.97	0.09
BMI (kg/m^2^)	20.48 ± 1.10	20.02 ± 1.46	0.39
VO_2_peak	26.50 ± 1.89^**^	19.86 ± 1.00	0.00
HR_rest_	73.33 ± 9.57	78.67 ± 13.92	0.29

*BMI, body mass index; VO_2_peak, maximal oxygen consumption; ^∗∗^*p* < 0.01.*

### Behavioral Performance

**FIGURE 3:**
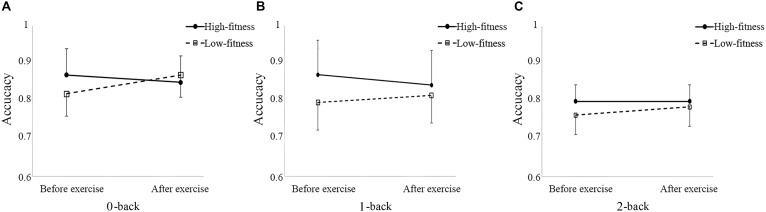
Illustration of the interaction effect in the accuracy of task performance between intervention conditions and fitness groups induced by acute aerobic exercise. Comparisons of the performance accuracy between the high- and low-fitness groups before and after exercise in the **(A)** 0-back task, **(B)** 1-back task, and **(C)** 2-back task.

In the 0-back task, a significant interaction effect of group ^*^ time was detected [*F*(1,22) = 4.95, *p* = 0.04,$\eta_{p}^{2}=0.18$]. Further *post hoc* analysis revealed that the accuracy was significantly better after exercise than before exercise in the low-fitness group [*F*(1,22) = 5.00, *p* = 0.04]. However, there was no significant difference between the accuracy before and after exercise [*F*(1,22) = 0.83, *p* = 0.37] in high-fitness group. We also examined the group difference in accuracy before and after exercise, which did not reveal any significant differences [before exercise: *F*(1,22) = 1.73, *p* = 0.20; after exercise: *F*(1,22) = 0.28, *p* = 0.60] (Figure 3).

In the 1-back task, we observed a significant interaction effect of group ^*^ time [*F*(1,22) = 5.06, *p* = 0.04,$\eta_{p}^{2}=0.19$]. Regarding the main effects, we found a marginally significant main effect of group [*F*(1,22) = 3.08, *p* = 0.09,$\eta_{p}^{2}=0.12$], while the main effect of time was not significant [*F*(1,22) = 0.24, *p* = 0.63]. The *post hoc* analysis revealed that the accuracy before exercise was slightly higher than that after exercise in the high-fitness group [*F*(1,22) = 3.75, *p* = 0.07], while there was no significant difference between the accuracy before and after exercise in the low-fitness group. Moreover, the accuracy in the high-fitness group was significantly greater than that in the low-fitness group before exercise [*F*(1,22) = 5.83, *p* = 0.03], while no difference was detected after exercise [*F*(1,22) = 0.86, *p* = 0.36].

In the 2-back task, no significant differences were observed in the main effects of group [*F*(1,22) = 2.34, *p* = 0.14] and time [*F*(1,22) = 0.79, *p* = 0.39]. Similarly, we did not observe and interaction effect of group ^*^ time [*F*(1,22) = 0.79, *p* = 0.39]. Regarding group differences, we did not observe any significant differences in accuracy before and after exercise for either fitness group (high-fitness group: [*F*(1,22) = 0.00, *p* = 1.00]; low-fitness group [*F*(1,22) = 1.57, *p* = 0.22]). Additionally, similar to that in the 1-back task, the accuracy in the high-fitness group was significantly greater than that in the low-fitness group before exercise [*F*(1,22) = 5.02, *p* = 0.04], but no significant difference was observed after exercise [*F*(1,22) = 0.43, *p* = 0.52].

Furthermore, we conducted independent sample *t*-tests before and after exercise to determine if there was any difference between the two groups in the accuracy of N-back task performance. The result indicated a significant difference in both groups before but not after exercise [before: *t*(11) = 2.315, *p* = 0.03; after: *t*(11) = 0.324, *p* = 0.749], which indicated that the high-fitness group had better accuracy than did the low-fitness group before exercise.

### Brain Imaging

Table 2 provides a statistical summary of the interaction effects and main effects during the 0-back, 1-back, and 2-back tasks.

**TABLE 2:** The Activated brain areas of participants with different cardiovascular functions at pre- and post-exercise in 0-back, 1-back, and 2-back task.

	**Peak activation**				
**Region**	***X***	***Y***	***Z***	***F-*value**	**Voxels**
Group ^*^ Time (0-back):					
R Cerebellum	24	−88	−42	20.53	138
**Time: (post–pre)**					
L Cerebellum	−12	−44	−54	21.65	42
**Group:(high–low)**					
L Inferior parietal lobule	−48	−30	40	15.13	26
Group ^*^ Time (1-back):					
R Cerebellum	46	−56	−52	20.49	37
**Time: (post–pre)**					
L Cerebellum	−10	−44	−52	18.51	112
R Cerebellum	10	−44	−56	18.61	63
R SMA	10	−22	70	16.35	16
R Medial temporal pole	58	16	−26	16.88	12
**Group: (high–low)**					
L Inferior parietal lobule	−52	−32	44	14.04	16
Group ^*^ Time (2-back):					
L Anterior cingulate cortex	−2	32	16	14.18	20
L Globus pallidus	−20	−6	−4	15.89	15
**Group: (high–low)**					
R Superior frontal gyrus	14	50	48	17.68	42

*Coordinates (mm) are in MNI space. L, left hemisphere; R, right hemisphere. *p* < 0.001 (uncorrected), *k* > 10.*

**FIGURE 4:**
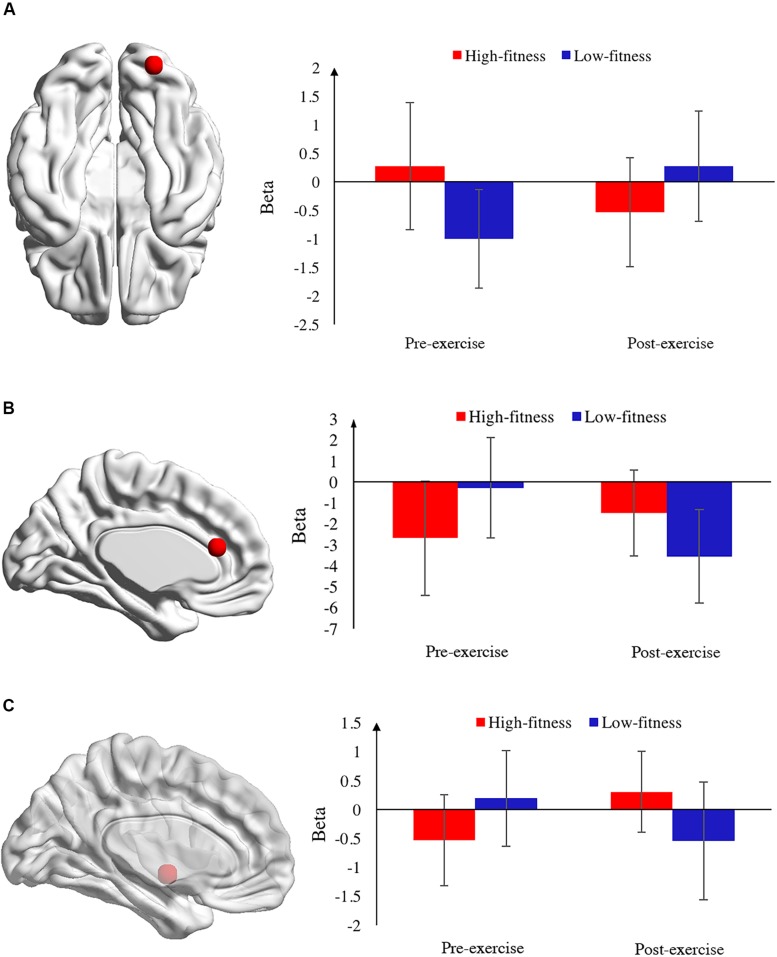
Localization and activation of brain regions during N-back tasks. **(A)** Activation of the right cerebellum in the 0-back task. The left panel presents the localization of the right cerebellum (RC) on an axial view. The red dot indicates the position of the RC. The right panel indicates the beta value of RC activation before and after exercise between the high- and low-fitness groups in the 0-back task. The red bar indicates high-fitness group and the blue bar indicates low-fitness group. **(B)** The activation of the left anterior cingulate cortex (ACC) in the 2-back task. The left panel presents the localization of the ACC on a sagittal view. The red dot indicates the position of the ACC. The right panel indicates the beta value of ACC activation before and after exercise between the high- and low-fitness groups in the 0-back task. The red bar indicates the high-fitness group and the blue bar indicates the low-fitness group. **(C)** The activation of the left globus pallidus (LGP) in the 2-back task. The left panel presents the localization of the LGP on a sagittal view. The red dot indicates the position of the LGP. The right panel indicates the beta value of LGP activation before and after exercise between the high- and low-fitness groups in the 2-back task. The red bar indicates the high-fitness group and the blue bar indicates the low-fitness group.

In the 0-back task, the results indicated that the right cerebellum (RC) was significantly activated in the interaction effect of time ^*^ group while the left cerebellum was activated in the main effect of time (Table 2). Considering the specific role of the RC under different conditions, we analyzed the RC as a region of interest (ROI) and computed the beta value of the RC. The activation of the RC between two time points was further examined by a paired sample *t*-test. The result indicated that the activation of the RC before exercise was slightly higher than that after exercise in the high-fitness group [*t*(11) = 1.93, *p* = 0.08] while the activation of the RC before exercise was relatively lower than that after exercise in the low-fitness group [*t*(11) = −3.88, *p* < 0.001]. Regarding the activation of the RC before exercise, an independent sample *t*-test was used to compare the difference between the two groups, which revealed that the activation of the RC in the high-fitness group was higher than that in the low-fitness group [*t*(22) = 3.11, *p* = 0.01]. Regarding the activation of the RC after exercise, the activation of the RC in the high-fitness group was lower than that in the low-fitness group [*t*(22) = −2.07, *p* = 0.05] (Figure 4). Additionally, we also examined the main effect of group, which revealed that the left inferior parietal lobule was greatly activated in high-fit group relative to low-fit group (Table 2).

Similarly, in the 1-back task, we also observed that the RC was activated in the interaction effect of group ^*^ time. Moreover, the main effect analysis revealed that the left inferior parietal lobule was activated in the main effect of group while the left cerebellum, RC, right auxiliary motor area, and right middle frontal gyrus were activated in the main effect of time. With regard to the activation of the RC in the interaction effect, the RC was also considered to be an ROI in the 1-back task to further compare the differential activation between conditions. It was found that the activation of the RC before exercise was higher than that after exercise in the high-fitness group [*t*(11) = 3.27, *p* = 0.01] while the activation of the RC before exercise was lower than that after exercise in the low-fitness group [*t*(11) = −2.07, *p* = 0.06]. The between-group analysis revealed that the activation of the RC in high-fitness group was lower than that in the low-fitness group after exercise [*t*(22) = −2.90, *p* = 0.01], while there was no significant difference in the activation of the RC between the high- and low-fitness groups before exercise [*t*(22) = 1.61, *p* = 0.12].

In the 2-back task, regarding the main effects, we found that the right superior frontal gyrus was activated in the group effect. The interaction effect analysis revealed that both the left anterior cingulate cortex (ACC) and the left globus pallidus (LGP) were activated in the interaction effect of group ^*^ time. The left ACC was then considered as an ROI in order to examine the differential activation under different conditions. Further ROI analysis revealed that the activation of the left ACC was higher after exercise than before exercise in the high-fitness group [*t*(11) = −1.87, *p* = 0.09], and lower after exercise than before exercise in the low-fitness group [*t*(11) = 3.74, *p* < 0.001]. The between-group analysis revealed that the activation of the left ACC in the low-fitness group was higher than that in the high-fitness group before exercise [*t*(22) = −2.30, *p* = 0.03], opposing results were observed after exercise [*t*(22) = −2.34, *p* = 0.03].

The LGP was another ROI that was analyzed to further examine within-group and between-group differences. A paired sample *t*-test indicated that the activation of the LGP after exercise was higher than that before exercise in the high-fitness group [*t*(11) = −3.00, *p* = 0.01), while no significant difference was found in the low-fitness group [*t*(11) = 1.64, *p* = 0.13]. Further analysis revealed that, similar to the activation of the left ACC, the low-fitness group exhibited higher LGP activation than did the high-fitness group before exercise [*t*(22) = −2.20, *p* = 0.04], while compared to low-fit group, the high-fitness group exhibited higher activation in the left LP [*t*(22) = 2.37, *p* = 0.03] (Figure 4).

## Discussion

In this study, we used functional MRI to detect fitness-dependent effect of cognitive performance immediately after acute aerobic exercise. There are several main findings: (1) at the behavioral level, significant interaction effects of group by time in accuracy were consistently detected in the 0-back and 1-back tests, which verified the hypothesis that there were significant differences in executive function between the high-fitness group and the low-fitness group after acute exercise. Furthermore, these findings indicated the moderating role of cardiovascular fitness in the effect of acute aerobic exercise on executive function. Additionally, the accuracy in the high-fitness group was significantly higher than that in the low-fitness group before exercise in the 1-back and 2-back tasks. (2) Similarly, at the neural level, we observed significant interaction effects of group by time in the 0-back, 1-back, and 2-back tasks, which demonstrated the neural mechanism underlying the moderating role of fitness in the effect of acute exercise on cognition. Regarding the interactive effect of group by time, it is plausible that dissociated neural correlates existed in this moderating effect of fitness when tasks of varying difficulty were performed.

At the behavioral level, we consistently observed significant interaction effects of group by time in the accuracy of the 0-back and 1-back tasks with two levels of difficulty. This confirmed our prediction that the mechanism underlying the effect of acute exercise on cognitive alteration is fitness-dependent, which indicated the potential moderating role of cardiovascular fitness in the effect of acute exercise on cognition. Although much attention has been afforded to exploring the cognitive benefits induced by acute exercise (Wei et al., 2013, 2014, 2017), it is largely unknown whether individual differences in fitness play a role in the beneficial outcomes of acute aerobic exercise. This study has extended the literature by exploring the neural mechanism of fitness as a moderator in the exercise-induced improvement of cognition using fMRI. The findings are also consistent with those of previous studies that observed differences in the performance of task-switching protocols in young adults with varying levels of fitness (Tsai et al., 2016). In another study investigating the effect of exercise variables on cognitive performance, it was also found that one session of moderate- to high-intensity exercise was associated with detrimental performance in the executive component of the Stroop task. This was particularly evident in lower-fitness individuals, which also supported the moderate role of fitness level in the effect of acute exercise on cognition (Labelle et al., 2014). Hotting and Roder (2013) reviewed the beneficial effects of physical exercise on neuroplasticity and cognition and addressed the importance of individual differences in responsiveness to exercise as indicated by cardiovascular fitness, and suggested that individual differences should be accounted for when inferring the relationships between exercise and cognition.

Additionally, we observed that the accuracy of the 0-back and 1-back tasks was improved after acute aerobic exercise compared to that before exercise in the low-fitness group. However, these improvements were not significant in the 1-back task and no such tendency was observed in the high-fitness group. It is reasonable to infer that the individuals with low fitness might be more responsive to acute exercise and therefore significantly benefited from the exercise. Comparatively, the high-fitness group did not benefit from acute exercise to the same extent as did the low-fitness group. Chang et al. (2014) found that individuals with high fitness levels had the longest response time in the Stroop incongruent condition, suggesting that high fitness was associated with poor performance for this measure of executive control. One explanation is that complex cognitive tasks recruit more cognitive resources, which allows individuals with high cardiovascular fitness to perform better in behavioral tasks. However, tasks of relatively low difficulty could not sufficiently mobilize the cognitive resources of high-fitness individuals. As a consequence, when we examined the changing trend in accuracy in the high-fitness group, this group’s performance was better in the 2-back task and poorer in the 0-back and 1-back tasks after exercise than before exercise.

Behavioral analysis of the groups revealed that the high-fitness group had significantly higher accuracy in the 1-back and 2-back tasks and higher accuracy in the 0-back task than that of the low-fitness group before exercise. This result demonstrated that the high-fitness group had better cognitive performance than that of the low-fitness group at baseline. However, such significant differences were not observed after exercise, which indicated that the behavioral difference between these two groups was reduced after exercise. The better performance of the high-fitness individuals at baseline indicated that cardiovascular fitness might induce improvements in executive function. Regarding the positive association between fitness and cognition, behavioral evidences are largely provided by investigations of age-related cognitive decline. A higher VO_2max_, an index of cardiovascular fitness, was found to be associated with better global cognitive function and better executive function (Freudenberger et al., 2016). Barnes et al. (2003) examined 346 old adults over the course of 6 years, and found that older participants with poorer cardiorespiratory fitness at baseline experienced greater cognitive decline over 6 years. This confirmed that cardiorespiratory fitness is associated most strongly with global cognitive function and attention/executive function (Barnes et al., 2003). Another 5-year follow-up study of 4,615 participants revealed that high levels of physical activity were associated with reduced risks of cognitive impairment and indicated the positive effect of fitness on cognitive performance (Laurin et al., 2001). An imaging study of 350 healthy adults also provided consistent findings on this topic. The study indicated that good fitness was associated with better cognitive performance as well as lower white matter hyperintensities (Boots et al., 2015). In a cohort study, it was found that high-fitness and normal weight was associated with better behavioral performance and later stages of electrophysiological indices of cognitive function (Song et al., 2016). Our result undoubtedly supported the beneficial effect of fitness on cognition, which implies the use of exercise for the prevention of cognitive decline with normal aging and as a treatment for cognitive impairment and certain neurological disorders associated with cognitive dysfunction, such as Parkinson’s disease and attention deficit hyperactivity disorder.

To examine the neural correlates underlying the effect of acute exercise on executive function moderated by fitness, we acquired the task-evoked blood oxygen level-dependent signal in all participants. The neuroimaging result revealed significant interaction effects of group by time in all N-back tasks in this study, which provided neurological evidence for the moderating role of cardiovascular fitness in the effect of acute exercise on cognition.

Intriguingly, we observed similar activation patterns in the 0-back and 1-back tasks. Specifically, both of these tasks were found to consistently activate the RC when we examined the interaction effects of group by time. The cerebellum is well-established as a key subcortical brain region that contributes to motor control and coordination; the cerebellum receives input from the sensory systems of the spinal cord and from other parts of the brain, and integrates these inputs in order to fine-tune motor activity. Cerebellar damage produces motor-related disorder of fine movement, equilibrium, posture, and motor learning in humans (Squire, 2014). Recently, increasing evidences have indicated that the cerebellum also plays an important role in cognition and learning. It was found to be involved in the voluntary shift of selective attention between sensory modalities (Akshoomoff and Courchesne, 1992) and goal-directed cognitive functions (Schmahmann, 1998). Notably, the dentate nucleus of the cerebellum/lateral cerebellar nucleus are crucial structures responsible for complex cognitive processing such as spatial navigation and working memory (Locke et al., 2018). Accumulating clinical evidences have demonstrated the potential cognitive deficits and personality changes associated with cerebellar disease, particularly schizophrenia, dementia, and other psychiatric disorders (Rapoport et al., 2000). Damage to the subcortical components of cerebellar circuits and non-motor areas of the cortex also caused higher-order cognitive deficits (Middleton and Strick, 2000). In this study, we consistently observed significant activation of the RC by examining the interaction effect of group by time, which confirmed that the RC plays a crucial role in the execution of executive function tasks of low and medium difficulty level. However, there is little evidence to demonstrate that this brain structure is associated with exercise-induced cognitive outcomes. In light of the findings that neuronal activity within cerebellar loops and areas of the prefrontal cortex is more related to aspects of cognitive function, it is plausible that acute exercise might alter the pattern of functional connectivity between cerebellar loops and cognition-related areas of the prefrontal cortex, and thereby further improve the performance of executive function after exercise. Such influence is also dependent on fitness level and exhibited a contrary trend between the two groups after exercise. The greater activation of the RC may be due to the greater benefit of exercise on the neural correlates underlying the improvement of executive function in the low-fitness group.

Moreover, similar activation was also detected in the inferior parietal lobule in both the 0-back task and the 1-back task when investigating group differences. A study of individuals with good fitness revealed decreased cerebral blood flow in the inferior parietal lobule after 10 days of exercise cessation, which indicated that the training-induced changes in blood flow in the inferior parietal lobule may be reversed with 10 days of exercise cessation (Alfini et al., 2016). It is likely that decreased activity of the inferior parietal lobule is associated with better fitness, because we also observed significantly decreased activation of the inferior parietal lobule in the high-fitness group compared to that in the low-fitness group after exercise. Accumulating evidences indicate the role of the inferior parietal lobule in sensorimotor integration, spatial attention, and visuomotor and auditory processing. However, it is unclear whether the inferior parietal lobule is involved in exercise-induced cognitive processing during low and medium difficulty tasks only, or whether it is also involved in processing during high difficulty tasks. This requires further investigation.

In contrast to the 0-back and 1-back tasks, the 2-back task exhibited a different activation pattern, which involved significant activation of the ACC after exercise, when we investigated the interaction effects of group by time. In the low-fitness group, the activation of the ACC was greater before exercise than after exercise, while the opposite was observed in the high-fitness group. This result indicated that the ACC could only be activated when performing complex information processing involving a higher cognitive load after exercise. It is well-established that the ACC is involved in the processing of cognitive control tasks (Botvinick et al., 2001), particularly in response to the conflicting stimuli in executive control tasks. It functions as a key area in the allocation of control resources based on the evaluation of the expected value of control (Shenhav et al., 2013). The decreased ACC activity in the low-fitness group might indicate that the demand of recruiting the ACC in conflict control was reduced, and, accordingly, the conflict control induced by exercise was improved. This finding implied that exercise might have improved cognitive function in the low-fitness group by decreasing the activity of the ACC. This finding is also consistent with that of previous study using a 2-back task that examined cognitive gains induced by acute exercise among female college students (Li et al., 2014). Moreover, Colcombe et al. (2004) found that those who exhibited greater cardiovascular fitness gain after exercise also had decreased ACC activity.

Similarly, we also detected significant activation of the LGP by examining the interaction of group by time in the 2-back task. Specifically, the high-fitness group exhibited greater activation of the LGP than did the low-fitness group after exercise. Moreover, this group exhibited greater activation of the LGP after exercise relative to that before exercise. These findings demonstrated that the LGP is another key brain region that exhibits exercise-induced activity that is affected by fitness level. Although the role of the LGP in such a moderating effect is unclear, increased LGP activity may be associated with improvement in the control of body movement in light of its connection to the cerebral cortex and the thalamic neuron network.

There are several limitations to this study. Regarding the differences in cardiovascular fitness between females and males in nature, we enrolled only females in this study in order to exclude the influence of sex as a confounding variable in the fitness-dependent effect of acute exercise on cognition. Although we confirmed such an effect in this study, we could not generalize the difference between the high-fitness group and the low-fitness group within females to the general population; this needs to be assessed in males in further investigations. Moreover, the small sample size might be another factor contributing to the non-significant behavioral difference between the high-fitness group and the low-fitness group in the 2-back task.

## Conclusion

In this study, we investigated the relevant neural mechanism underlying the fitness-dependent effect of acute exercise on executive function. The main findings partly verified our hypothesis, in that we detected such an effect in the 0-back and 1-back tasks. The results indicate that the RC is a key brain region in the processing of simple executive tasks following acute aerobic exercise, while the subcortical areas played a dominant role in the processing of relatively complex executive tasks after exercise. The neural evidence supports the fitness-dependent effect of acute exercise on cognitive performance from cognitive neuroscience prospective.

## Data Availability

All datasets generated for this study are included in the manuscript and/or the supplementary files.

## Ethics Statement

The studies involving human participants were reviewed and approved by the Institutional Review Board of East China Normal University. The patients/participants provided their written informed consent to participate in this study.

## Author Contributions

G-XW and LL: conceptualization, project administration, and funding acquisition. LL, MF, SZ, JC, and G-XW: methodology. JC, XW, MF, G-XW, and SZ: formal analysis. JC and LL: investigation. LL, G-XW, and MF: data curation. G-XW, L-ZC, LL, and SZ: writing original draft preparation. SZ and G-XW: writing review and editing. L-ZC: visualization. G-XW: supervision.

## Conflict of Interest Statement

The authors declare that the research was conducted in the absence of any commercial or financial relationships that could be construed as a potential conflict of interest.

## Footnotes

1 http://www.fil.ion.ucl.ac.uk/spm/software/spm12/
